# Formulating Sustainable Emulsions: Mandelic Acid and Essential Oils as Natural Preservatives

**DOI:** 10.3390/molecules29184510

**Published:** 2024-09-23

**Authors:** Jana Pavlačková, Pavlína Egner, Pavel Mokrejš, Magda Janalíková

**Affiliations:** 1 Department of Fat, Surfactant and Cosmetics Technology, Faculty of Technology, Tomas Bata University in Zlín, Vavrečkova 5669, 760 01 Zlín, Czech Republic; pavlackova@utb.cz; 2 Department of Polymer Engineering, Faculty of Technology, Tomas Bata University in Zlín, Vavrečkova 275, 760 01 Zlín, Czech Republic; mokrejs@utb.cz; 3 Department of Environmental Protection Engineering, Faculty of Technology, Tomas Bata University in Zlín, Vavrečkova 275, 760 01 Zlín, Czech Republic; mjanalikova@utb.cz

**Keywords:** cosmetic matrix, mandelic acid, essential oil, TEWL, hydration, antimicrobial effect, natural preservative

## Abstract

Emulsion products with natural antimicrobials are becoming increasingly popular for topical application. *Mandelic Acid* is interesting in cosmetics due to its potent exfoliating properties, which have driven advancements in skincare technologies. Essential oils have various properties, of which the most useful in cosmetics are those that do not cause irritation, smell pleasant, and have other beneficial properties such as antimicrobial effects. Emulsions with *Mandelic Acid* and essential oils from *Satureja montana*, *Lemongrass*, and *Litsea cubeba* were formulated and microbiologically tested for their preservative effectiveness. The effect of the treatments on skin condition was monitored by non-invasive diagnostic methods, such as hydration, transepidermal water loss, and pH value. Sensory analysis revealed that the matrix containing *Mandelic Acid* alone or combined with *Litsea Cubeba Oil* was the best-performing formulation, consistent with the compliant results of antimicrobial efficacy. The topical form of this cosmetic product has demonstrated excellent preservative activity and desirable biophysical efficacy on the skin.

## 1. Introduction

The cosmetics industry is a dynamic sector driven by constant consumer demands for effective and innovative yet safe products. So nowadays, there is an effort to produce cosmetics, whether with an emulsion or a gel base, with natural ingredients as a response to the preferences of today’s consumers who choose a sustainable and healthy lifestyle [1]. Preservatives need to be included when developing a safe cosmetic product. Conventional preservatives often raise concerns due to possible adverse effects on skin and overall health. Striking a balance between the need to preserve the product and ensure its safety for the consumer remains a long-term and demanding process. As a result, alternative preservation methods are increasingly being explored. The process consists of two main phases: the formulation and testing of the toxicity and microbial contamination of the ingredients to be considered when selecting raw materials. The presence of banned or restricted substances that may pose a significant risk to consumers must meet the requirements according to regional valid legislation on cosmetic products [2]. For example, the current legislation governing cosmetic products within the European Union (EU) is established by Regulation (EC) No 1223/2009 of the European Parliament and of the Council on cosmetic products [3]. This regulation defines requirements on the safety, labelling, distribution, and traceability of cosmetic products within the EU market. The properties of the topical cosmetic product are determined by the purpose of use, which is also important for the concentration of the active antimicrobial agents. Higher concentrations of active antimicrobials are chosen for the formulation of antimicrobial cosmetics, and lower concentrations for ordinary cosmetics. According to that regulation, preservatives are substances that are exclusively or mainly intended to inhibit the growth of microorganisms in cosmetic products, and they are listed among preservatives allowed in cosmetic products (Annex V) [3].

To ensure consumer safety, it is necessary to protect the cosmetic product by creating a preservation system from the inside (regarding formulation) and from the outside (regarding packaging). The microbial contamination of the product can occur during manufacture; this is known as primary contamination. It can be avoided by strict adherence to ISO 22716:2007 Cosmetics—Good Manufacturing Practices (GMP) principles of the microbial control of raw materials, disinfection of equipment, and involvement of qualified personnel in reducing the risk of contamination [4]. Another potential risk of the secondary contamination of the cosmetic product is the consumer who uses the product. Here, the packaging, shape, material, and dosage system of the cosmetic product play a significant role [5].

Water is one of the crucial growth factors for microorganisms and, at the same time, an essential component of cosmetic products. Therefore, a microbiological analysis of cosmetic product batches is necessarily embedded in the guidelines [6]. Natural skin and mucous membrane barriers are an effective defense mechanism against microbial attack [7]. If this barrier is compromised, for example, by cosmetic products, the risk of microbial infection increases, particularly around the eyes, mucous membranes, and compromised skin. This susceptibility is more pronounced in children under three years of age, the elderly, and individuals with compromised immune systems. Products applicable in this way in this group are explicitly defined in the ISO 17516:2014 as Category 1, while Category 2 includes all other cosmetic products [8]. According to that standard, a qualitative test of a cosmetic product should prove the absence of these opportunistic pathogens—*Escherichia coli*, *Pseudomonas aeruginosa*, *Staphylococcus aureus*, and *Candida albicans*—in 1 g or 1 mL of a cosmetic product of Category 1 or in 0.1 g or 0.1 mL of a cosmetic product of Category 2. Quantitative limits are determined by the total viable count for mesophilic microorganisms: 10^2^ CFU/g (CFU/mL) in Category 1 and 10^3^ CFU/g (CFU/mL) in Category 2 [8].

Amongst the most common microorganisms causing infections from cosmetic products are the bacteria *Pseudomonas aeruginosa*, *Enterobacter* sp., *Staphylococcus aureus*, and, less frequently, *Burkholderia cepacia*, and filamentous fungi such as *Aspergillus* spp. [9]. On the other hand, the range of microbes found in cosmetics contains far more species such as *Achromabacter xylosoxidans*, *Bacillus firmus*, *Citrobacter freundii*, *Corynebacterium* spp., *Enterococcus faecium*, *Pluralibacter gergoviae*, *Klebsiella oxytoca*, *Klebsiella pneumoniae*, *Micrococcus* spp., *Paenibacillus* spp., *Pantoea agglomerans*, *Pseudomonas putida*, *Rhizobium radiobacter*, *Serratia marcescens*, and *Staphylococcus* spp. in terms of bacteria, and even yeasts such as *Candida albicans* or the fungi *Bjerkandera* spp., *Cladosporium* spp., *Peniphora* spp., or *Trametes* spp. [10,11].

In recent years, consumers have shifted their interest towards natural cosmetics, to which the cosmetics industry has responded by reducing synthetic preservatives, developing preservative-free cosmetics, or developing so-called self-preserving cosmetics. Self-preservation or free preservation is preservation without the use of an additional chemical ingredient classified as a preservative in the annexes of the cosmetic legislation [2,12]. There is a drive to replace synthetic antimicrobials with natural substances [13]. Parabens are the most abundant of the synthetic substances and are used either in a mixture of up to 0.8% or up to 0.4% individually [3]. Methylparaben and propylparaben are most common in cosmetic products [14,15]. Their antimicrobial activity is well referenced. These substances have undergone several reviews, with the most recent occurring in 2019, during which the CIR Expert Panel recognized parabens as safe [16]. Other synthetic preservatives include *Methylisothiazolinone* (e.g., Kathon, Euxyl K 100), *Quaternium-15*, *Imidazolidinyl Urea*, *2-bromo-2-nitro-propane-1,3-diol* (e.g., Bronopol), *Triclocarban*, *Triclosan*, *Benzyl alcohol*, *Chlorhexidine*, *Iodopropynyl butylcarbamate*, or *Phenoxyethanol* [3].

Essential oils (EOs), as the natural plant mixtures of dozens of substances, have been long recognized for their antibacterial, antifungal, antiviral, insecticidal, and antioxidant properties [17,18]. It is crucial to know the safety profile of essential oils to maximize their beneficial effects while minimizing the risk to the user. Due to the chemical complexity of essential oils, it is difficult to investigate which individual component is responsible for certain adverse effects [19]. The comparable properties were observed among some aromatic acids of natural origin, especially *Mandelic Acid* [20,21,22]. Thus, these natural compounds, like purified plant extracts or essential oils, appear to be an acceptable natural alternative to achieve microbial purity for the product [23]. However, most of these substances, although they have been shown to have antimicrobial activity, do not qualify as preservatives under current legislation [3]. A substantial number of published experiments enclosed a determination of antimicrobial activity of pure essential oils [24,25], e.g., from *Lemongrass* [26] or *Litsea Cubeba* [27], while the incorporation of these natural preservatives into the cosmetic formulation is far less studied [28,29,30].

The study aimed to prepare a cosmetic emulsion product based on natural ingredients preserved with essential oils in combination with *Mandelic Acid*, and to determine its antimicrobial activity and biophysical properties in vivo.

## 2. Results

### 2.1. Antimicrobial Activity of Essential Oils

Essential oils (EOs) from *Satureja*, *Lemongrass,* and *Litsea* were tested against Gram-positive and Gram-negative bacteria and yeasts. The antimicrobial activity of essential oils alone was assessed using the disk diffusion method, with the results summarized in Table 1. *Satureja Montana* and *Lemongrass* EOs exhibited similar antimicrobial activity against all tested microorganisms except *P. aeruginosa*. Additionally, *Litsea Cubeba* EO was ineffective against the yeast *Candida albicans*. In our previous work [31], 5 wt% *Mandelic Acid* was shown to inhibit *P. aeruginosa*, *Micrococcus luteus*, *S. aureus*, *E. coli*, *C. albicans,* and *C. parapsilosis* by the disk diffusion method.

### 2.2. Antimicrobial Effectiveness Test

**Figure 1:**
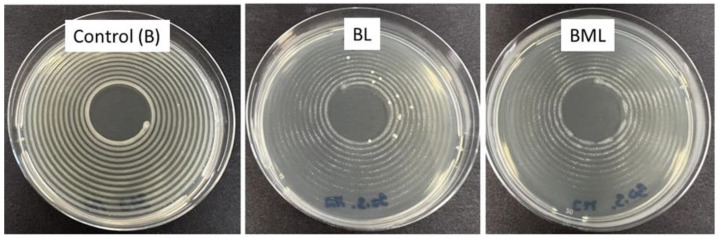
Plating of *P. aeruginosa* after 7 days of storage within samples: B—control, BL—base emulsion with *Litsea Cubeba Oil*, and BML—base emulsion with *Mandelic Acid* and *Litsea Cubeba Oil*.

The results of the antimicrobial effectiveness test are summarized in Table 2. Total viable counts of *S. aureus* and *P. aeruginosa* were evaluated after 7, 14, and 28 days of room storage. Live bacteria of *S. aureus* were only found in the base emulsion (B) and base emulsion with *Lemongrass Oil* (BG) samples after 7 days; no persistent bacteria were detected in any other sample or measurement day. Interestingly, all samples containing *Mandelic Acid* (M) were free of *S. aureus*. On the other hand, *P. aeruginosa* was less sensitive to the tested antimicrobials. As a control without the antimicrobial agent, the B sample contained these Gram-negative bacteria during the whole storage time, up to 7 Log orders. The base emulsion with *Litsea Cubeba Oil* (BL) sample inhibited *P. aeruginosa* by 3 Log orders after 7 days compared to the control B sample (Figure 1). Nevertheless, the difference decreased to a little more than 1 Log order later. The base emulsion with *Satureja Montana Oil* (BS) and BG samples were free of microorganisms during the whole storage time. Thus, *Satureja Montana* and *Lemongrass* EOs seemed highly effective in cosmetic product protection. The base emulsion sample with *Mandelic Acid* (BM) reduced *P. aeruginosa* up to the detection limit of the method used during 28 storage days. Surprisingly, adding *Satureja Montana* EO and *Mandelic Acid* (BMS) samples was insufficient to inhibit *P. aeruginosa.* The base emulsion with M and *Lemongrass Oil* (BMG) samples decreased the *P. aeruginosa* population to 3.5 log orders after 7 days, up to the detection limit after 14 days, and remained clear after 28 days. The base emulsion with *Mandelic Acid* and *Litsea **C**ubeba Oil* (BML) samples, in comparison with the BL sample (Figure 1), successfully reduced both bacteria during 28 storage days, and thus, it represents the best result of this challenging experiment.

### 2.3. In Vivo Efficiency

**Figure 2:**
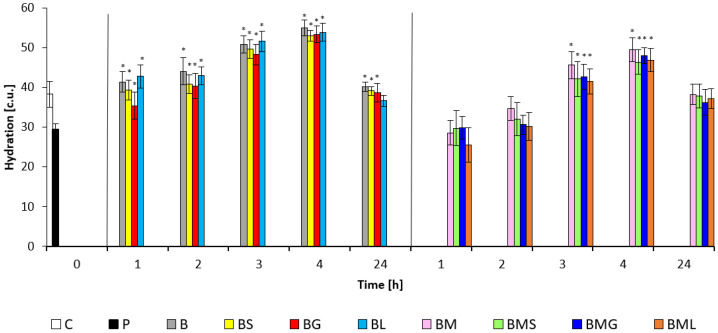
Hydration potential of emulsion matrices: C—control; P—SDS pretreatment; B—base emulsion; BS—base emulsion with *Satureja Montana Oil*; BG—base emulsion with *Lemongrass Oil*; BL—base emulsion with *Litsea Cubeba Oil*; BM—base emulsion with *Mandelic Acid*; BMS—base emulsion with *Mandelic Acid* and *Satureja Montana Oil*; BMG—base emulsion with *Mandelic Acid* and *Lemongrass Oil*; BML—base emulsion with *Mandelic Acid* and *Litsea Cubeba Oil*; * indicates statistically significant difference vs. SDS pretreatment (*p* < 0.05).

The biophysical parameters of hydration, TEWL, and pH values quantify the function level of the skin barrier. The skin hydration status after applying the prepared emulsion matrices is presented in Figure 2. Higher hydration values were observed in the areas of the volar side of the forearm treated with the B sample and the B with EOs after SDS pre-treatment than in the skin with the BM samples with or without EOs. Hydration values were above 40 c.u. for the B samples with EOs two hours after application. Similar skin hydration values treated with the BM samples with or without EOs samples were measured with an hour delay compared to emulsions without M. Four hours after skin exposure to all tested formulations, the highest moisturizing activity was significant (*p* < 0.05). The difference between the first and the fourth hour results was about 10 c.u. Almost identical values of the hydration effect were measured after 24 h of exposure to all emulsion matrices. Among emulsions with EOs, the highest hydration level was recorded for the baseline matrix and then the BL sample after the first four hours. The essential oils incorporated into the base formulations did not cause a statistically significant difference in the level of skin moisturizing. Overall, there was a meaningful improvement in skin recovery according to the scale described in Section 4.6.3.

**Figure 3:**
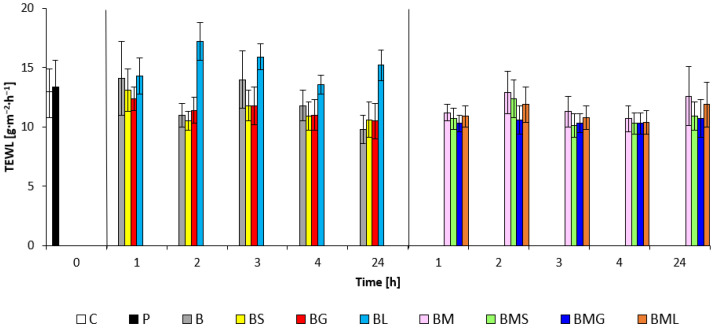
Barrier potential of emulsion matrices: C—control; P—SDS pretreatment; B—base emulsion; BS—base emulsion with *Satureja Montana Oil*; BG—base emulsion with *Lemongrass Oil*; BL—base emulsion with *Litsea Cubeba Oil*; BM—base emulsion with *Mandelic Acid*; BMS—base emulsion with *Mandelic Acid* and *Satureja Montana Oil*; BMG—base emulsion with *Mandelic Acid* and *Lemongrass Oil*; BML—base emulsion with *Mandelic Acid* and *Litsea Cubeba Oil*; No statistically significant difference vs. SDS pretreatment (*p* < 0.05) was found.

The application of the EO formulation series with and without M on the skin did not violate the integrity of the *stratum corneum*, as was proved by TEWL values that were not above the scale range given in Section 4.6.3. At the observed times, TEWL values fluctuated in the treated areas (Figure 3); for samples of the basic matrix, including added EOs, the values were from 9.8 to 17.2 g·m^−2^·h^−1^ and for samples with M and EOs the values ranged from 10.3 to 12.9 g·m^−2^·h^−1^. A statistically significant difference was not found between any sample compared to SDS pre-treatment. The highest skin water loss was observed after BL treatment. Nevertheless, even these values correspond to the healthy function of the skin barrier.

**Figure 4:**
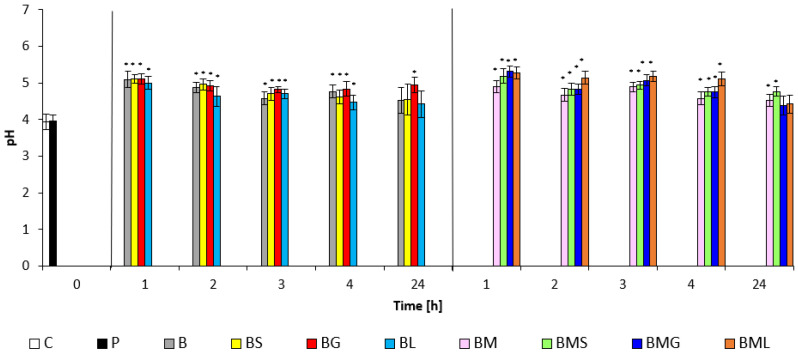
pH values of emulsion matrices: C—control; P—SDS pretreatment; B—base emulsion; BS—base emulsion with *Satureja Montana Oil*; BG—base emulsion with *Lemongrass Oil*; BL—base emulsion with *Litsea Cubeba Oil*; BM—base emulsion with *Mandelic Acid*; BMS—base emulsion with *Mandelic Acid* and *Satureja Montana Oil*; BMG—base emulsion with *Mandelic Acid* and *Lemongrass Oil*; BML—base emulsion with *Mandelic Acid* and *Litsea Cubeba Oil*; * indicates statistically significant difference vs. SDS pretreatment (*p* < 0.05).

The effect of the prepared matrices on the skin pH is presented in Figure 4. The initial acidity of the skin mantle compared to the control site and the site treated with SDS solution was adjusted from pH values of about four by the application of prepared matrices with EOs after the first hour in the pH range of 5.09–5.11; during further monitoring at the specified times, a decrease in the pH range to 4.42–4.96 was recorded. A similar result was found in matrices with the addition of M and EOs in the range of 4.42–5.31. Physiological pH values were measured after 24 h of the skin test.

### 2.4. Sensory Analysis

The ordinal test assessing spreadability was able to show that there were differences between the samples at the 0.05 significance level. Based on the sum of the rankings, the BM sample was ranked as the most spreadable, the same as the BML sample (Table 3). The spreadability of the base matrix B sample, which was the most viscous by visual assessment, was ranked as the least comfortable. The assessors perceived the emulsion matrices with the addition of M to have more sensory pleasure and better spreadability. This difference was not supported by the results of the preference ranking test and the paired absorbency test, which failed to demonstrate a difference with 95% confidence between the range of samples assessed and between pairs of samples.

## 3. Discussion

### 3.1. Cosmetic Matrix Type and Composition

The primary task was to decide which kind of matrix is suitable for applying to the skin. The choice of a suitable matrix depends on the possible hydration potential and the concentration of the active substance used. Topically applied preparations, which are expected to have a short-term moisturizing effect on the *stratum corneum*, are formulated with a higher free water content as an oil-in-water emulsion (*o*/*w*) or can form a gel matrix [32]. The occlusive effect of o/w emulsions and gels can be modified by adequate viscosity regulators, while the structure can also be reflected in the magnitude of the barrier effect. On the other hand, in vehicles with a planned long-term moisturizing effect, there should be a higher lipid content with a lower proportion of the aqueous component formulated as a water-in-oil emulsion (*w*/*o*) [33,34]. This basic formulation difference in matrices manifests in more efficient hydration in the emulsion system *w*/*o* [35,36], but such a system tends to be less sought after from a cosmetological point of view. The additives determining the character of the matrix and the selected active ingredients can be so diverse that they may no longer be decisive for the effectiveness of the final product according to the selected vehicle [32]. In the decision-making process regarding the performance properties of the final product, the *o*/*w* emulsion was chosen as the basic matrix for the above reasons, the composition of which is presented in Table 4.

The secondary task focused on the preparation of the selected topical matrix containing sustainable natural ingredients. A natural preservation system was selected to ensure the microbial stability of the product. For this purpose, EOs supported by the effect of M were a suitable selection since the microbial inhibition by these substances has been described previously [31]. It is known that the chemical composition of EOs is significantly influenced by their origin, location, time of harvest, and processing conditions. Their antibacterial activity is based on their chemical composition (e.g., limonene, carvacrol, p-cymene, geraniol, or neral). However, each component can play a key role not only alone, but also in interaction with other major or minor components [37].

However, these compounds (EOs) are not listed in Annex V of Regulation (EC) No 1223/2009 among the preservatives allowed in cosmetic products [3]. Preservatives are of major importance in fighting microbial contaminations of cosmetic forms. *Mandelic Acid* is used in treating skin problems (acne, etc.), and for its unique anti-aging activity [38]. Because M is a large-form molecule, it is much slower to penetrate the skin layer, allowing for more even treatment. This slow penetration makes it much gentler on the delicate lower *dermis* and it can be used as a skin care treatment on even delicate skin. Sunflower oil, due to its oleic and linoleic acid content, is a suitable ingredient in skin care products. It has been shown to preserve the integrity of the *stratum corneum* and promote skin hydration without causing erythema [39,40]. *Butyrospermum parkii*, which is characterized by its higher melting point and semi-solid consistency, also optimizes skin properties through the mechanism of relipidation [41]. *Cera Alba* has a similar effect on the skin, and its antimicrobial activity is also known but marginally verified. Its emulsifying abilities cannot be overlooked, modifying products’ physical and sensory properties, especially their adhesion to the skin [42]. Another of the used emollients, *Theobroma Cacao (Cocoa) Seed Butter*, provides broad-spectrum protection from UV-A and UV-B radiation effects along with endogenous photoprotection owing to its high antioxidant and anti-inflammatory properties. *Cocoa Butter* works as an excellent stabilizer for several applications related to cosmeceuticals [43]. *Ricinus Communis Seed Oil* is rich in unique hydroxy fatty acids. It is included in cosmetic formulas as a solvent, and it acts as an emollient due to its thick consistency [44]. Thanks to such efficacy, there was no disruption of the healthy functioning epidermal barrier according to the monitored water loss values in vivo testing of prepared formulation samples. The irritation of the *stratum corneum* with a higher TEWL value is evident from Figure 3 compared to the control. Olivoil Avenate Emulsifier is suitable for sensitive and delicate skin. Furthermore, it is useful to build up the anisotropic lamellar phase *o*/*w* from natural ingredients [45]. It gives a very pleasant white appearance and characteristic soft touch to emulsions, which was confirmed by the sensory analysis results of the assessed samples.

### 3.2. Antimicrobial Effectiveness of Cosmetic Emulsion

Mandelic acid is a hydroxy-acid active against some bacteria such as *S. aureus*, *K. pneumoniae*, and *P. aeruginosa* [21,46,47]. In the study [48], the antibacterial and antifungal activity of *Mandelic Acid* in ointments was examined, and its preservative properties were proved to be sufficient, as in the present study. Another long-term study of prepared gels and lotions with 2% and 10% concentrations of *Mandelic Acid* also highlights its conclusive antibacterial effects and other benefits for problematic skin [49]. Essential oils are natural mixtures of overly complex structures, chemically derived from terpenes and their derivatives. An important property of EOs is the ability to kill bacterial cells by disrupting cell structures [25]. The antimicrobial activity of *Satureja Montana Oil* was evaluated as the most effective among the five selected essential oils against seven tested microorganisms: *Pseudomonas aeruginosa*, *Streptococcus pyogenes*, *S. mutans*, *S. sanguis*, *S. salivarius*, *Enterococcus faecalis*, and *Lactobacillus acidophilus* [50].

It was widely observed that Gram-positive bacteria are more sensitive to the influence of essential oils than Gram-negative bacteria because of the simpler arrangement of the cell wall [51,52]. The antibacterial effect of essential oils and their constituents is linked to their lipophilic nature, which allows them to accumulate in membranes and thus act on their destruction [52]. Some studies suggest that conventional preservatives are replaced by the antimicrobial efficacy of some essential oils [53,54]. By ensuring microbiological safety, the consumer is protected from possible pathogenic microorganisms, while the physical and chemical properties of the product must not be degraded. In this study, the BML emulsion, containing *Mandelic Acid* and *Litsea Cubeba Oil*, has been shown to have an excellent microbiological stability, and it is suitable to replace the use of synthetic preservatives in this kind of formulation. Antimicrobial protection is also influenced by the acidity of the skin mantle. Degreased skin and skin treated with the model formulations showed low pH values corresponding to the slightly acidic surface of the skin [55].

### 3.3. In Vivo Biophysical Parameters of Cosmetic Emulsion

Pre-treatment with SDS solution, as a surfactant, is used in numerous studies [34,56,57] to standardize the initial characteristics of the skin adequately to routine skin care, e.g., showering with shower gels with surfactants. Surfactants negatively affect the structural and functional integrity of the skin, alkalize the skin surface, and remove lipids and proteins [58].

The selected EOs did not significantly contribute to the overall hydration activity of the basic matrix or the matrix with the addition of M, but there is a noticeable difference in hydration level. Samples containing *Mandelic Acid* moisturized the skin less. The humectants *Glycerin* [35] and *Aloe Barbadensis Extract* [59] are present in the formulation, which has been verified by studies not only for its moisturizing effect but also for its regenerative effect [60,61]. *Glycerin* is one of the most frequently used ingredients in the cosmetic industry due to its ability to bind water. In addition, it can also optimize the humidity of the product itself [62]. *Aloe Barbadensis Extract* is a rich source of bioactive compounds such as polysaccharides, antioxidants, vitamins, and minerals. Its ability to retain water in the *stratum corneum* is also evidenced by the presence of the amino acids histidine, arginine, threonine, serine, glycine, and alanine [63]. In addition to the active ingredients, the functional ingredients also had an influence on the hydration potential of the prepared formulation. Most formulated cosmetic products contain ingredients with multifunctional potential. The observed moisturizing efficacy of formulations with EOs was comparable to the moisturizing efficacy of the basic formulation without these ingredients (B). The addition of MA causes a decrease in moisturizing efficacy within 24 h after application (see Figure 2). A certain moisturizing effect can also be attributed to emollients present in the composition of emulsion matrices, where they fundamentally affect the user’s perception of differences in the consistency and texture of the final product after application to the skin. A number of ingredients, such as *Butyrospercum Parkii*, *Cera Alba*, *Cocoa Butter*, and *Ricinus Communis Seed Oil*, in the cosmetic formulation act against water loss through the outermost layer of the skin, thus helping natural skin regeneration [44]. Studies [64,65] state that skin pH is affected by occlusion and the usage of cosmetic products, as well as the state of the skin’s natural microbiome and age. The prepared formulations caused quite a rapid shift in pH, which remained almost unchanged in the monitored intervals. A low variability of pH values of the skin surface was observed after the application of model formulations, which could be explained comparably to the study [66] by the stabilization of the skin surface pH by applying a slightly acidic emulsion. Similar changes in pH values are reported in a study examining the effect of various vehicles with panthenol [67]. The model base formulation and its modifications can be evaluated as capable of improving or keeping the skin of volunteers in a healthy condition.

### 3.4. Sensory Analysis of Cosmetic Emulsion

Consumers prefer *o*/*w* emulsions and gels that are less sticky and greasy, easier to spread, and better absorbed. The organoleptic properties of skin care products are determined by the ingredients used in the formula, such as viscosity regulators, fillers, emulsifiers, and others. The most common solvent is water, followed by emollients between 3 to 20 wt%. Due to their diverse chemical structure, which determines their physico-chemical properties, the consumer perceives them after application to the skin with different sliding properties, reducing friction and modifying the product’s spreadability. Their effect is very often described as softening or smoothing [68,69]. When considering the organoleptic properties and sustainability of the prepared emulsion formulations, from the user’s perspective, matrices containing *Mandelic Acid* and essential oils, offering both emollient effects and antimicrobial activity, were perceived as more comfortable.

## 4. Materials and Methods

### 4.1. Chemicals and Microorganisms

The raw materials to produce 100 g of the basic emulsion matrix (B) and its modifications including *Mandelic Acid* (M) and essential oils (EOs) are listed in Table 4, where the INCI name, concentration, function, and supplier are given. The composition of tested essential oils is shown in Tables S1 and S2.

All microorganisms (*Cutibacterium acnes* CCM 7417; *Escherichia coli* CCM 3954; *Klebsiella aerogenes* CCM 2531; *Klebsiella oxytoca* CCM 2934; *Pseudomonas aeruginosa* CCM 3955; *Staphylococcus aureus* CCM 3953; and *Candida albicans* CCM 8276) were obtained from the Czech Collection of Microorganisms (CCM, Czech Republic). All microbiological media—nutrient agar, Mueller Hinton agar (MHA), reinforced clostridial agar (RCA) and Sabouraud agar (SA)—were supplied by Himedia Laboratories Pvt. Ltd., Mumbai, India.

### 4.2. Disk Diffusion Method

The disk diffusion method was used to determine the antimicrobial activity of the pure essential oils used in this study [70]. Briefly, the sterile 6 mm disks (Whatman, Maidstone, United Kingdom) impregnated with 10 μL of EO (*Satureja Montana Oil*—S, *Lemongrass Oil*—G, *Litsea Cubeba Oil*—L) were placed on plates inoculated with a 0.5 McF turbid suspension of bacteria (*S. aureus*, *E. coli*, *P. aeruginosa*, *K. oxytoca*, and *K. aerogenes*) on MHA, while *Cutibacterium acnes* bacteria were on RCA and the suspension of yeasts (*Candida albicans*) was on SA. The plates were incubated aerobically at 37 °C/24 h for bacteria (except plates with *C. acnes*, which were incubated anaerobically) and at 25 °C/5 days for yeasts. The diameters of the inhibition zones were measured, including the paper disk. All experiments were repeated three times.

### 4.3. Preparation of Model Emulsion Matrices

For the experiment, a base emulsion matrix was prepared and divided in half: one was left without (base emulsion, B), and to the other 5 wt% *Mandelic Acid* was added (BM). To the B matrix, 0.2 wt% EOs of three types were individually added (BS—*Satureja Montana Oil*, BG—*Lemongrass Oil*, and BL—*Litsea Cubeba Oil*), and similarly, they were incorporated into the BM matrix (BMS, BMG, and BML).

The basic emulsion matrix was made by weighing the aqueous and oil phase raw materials (Table 4) into a beaker, which was then heated in a water bath to a temperature of approximately 65 °C. The aqueous phase was stirred into the oil phase using a Heidolph RZR 2020 homogenizer (IKA, Staufen, Germany) at 2000 rpm for about 10 min, i.e., until the entire mixture was cooled. In the case of the emulsions containing *Mandelic Acid*, the procedure started by adjusting the pH of the *Mandelic Acid* solution (5 wt% of the total weight) using a 30 wt% NaOH solution to a pH value of 5.5, and then it was added to the aqueous phase. In the case of EOs (0.2 wt% of total weight), these were mixed to the oil phase and both phases were heated to about 65 °C (see above). The pH of the prepared emulsion was always about 5.5–6.0.

### 4.4. Antimicrobial Properties

To determine the antimicrobial effectiveness of the fabricated cosmetic products, bacterial suspensions of *Staphylococcus aureus* with a density of 3.47 × 10^7^ CFU/mL and *Pseudomonas aeruginosa* with a density of 1.63 × 10^6^ CFU/mL were prepared. A total of 1 g of each emulsion (B, BS, BG, BL, BM, BMS, BMG, and BML) was inoculated with 10 μL of either *S. aureus* or *P. aeruginosa* bacterial suspension. Each sample, in a glass tube, was mixed thoroughly and incubated in the dark at 22 ± 2 °C. The total viable counts were performed by the plate count method on nutrient agar, diluting the sample in saline solution using the automatic spiral plater Eddy Jet 2W (IUL, Barcelona, Spain) at 7, 14, and 28 days after inoculation. The plates were incubated at 37 °C/24 h. The colonies were counted by an automatic colony counter with the software Sphere Flash version 1.0.1.6 (IUL, Barcelona, Spain) and expressed as Log CFU/g. The detection limit was 2 × 10^2^ CFU/g.

### 4.5. Organoleptic Properties

The assessment of the organoleptic properties of the prepared emulsion formulations was carried out by a panel of 13 assessors trained according to ISO 8586:2023 [71], who were acquainted with the course and goal of the sensory evaluation. The evaluation conditions were ensured according to the requirements specified in ISO 6658 [72] and ISO 8589 [73]. The samples were presented randomly at a controlled temperature of 22 ± 1 °C under normal lighting conditions in a sensory laboratory equipped with sensory cubicles.

The sensory assessment questionnaire included two ranking tests [74] and one paired comparison test [75]. The ranking test investigated the spreadability of eight emulsion formulations (1—the best spreadable sample, 8—the least spreadable sample) and the preference for the emulsions (1—the most preferred sample, 8—the least preferred sample). A paired comparison test for the absorption of samples applied to the skin was performed with pairs B and B with the addition of *Mandelic Acid* (BM); B with the addition of S*atureja Montana Oil* (BS); B with the addition of *Cymbopogon Schoenanthus Oil; Lemongrass* (BG) and B with the addition of *Mandelic Acid* and *Cymbopogon Schoenanthus Oil* (BMG); and B with *Litsea Cubeba Oil* (BL) and B with the addition of *Mandelic Acid* and *Litsea Cubeba Oil* (BML).

### 4.6. Skin Diagnostics

The effect of the prepared emulsion matrices on the skin of the volar side of the forearm was monitored using non-invasive bioengineering instrumental methods quantifying the biophysical parameters of the skin: hydration, transepidermal water loss (TEWL), and pH.

#### 4.6.1. Volunteers

Eight women volunteers aged 30 to 45 (43.5 ± 2.6) without any health problems participated in the skin diagnosis. The volunteers were familiar with the goal of the experiment and its progress. Nevertheless, the selection of volunteers and the testing procedure were in accordance with international ethical principles of biomedical research with human participants [76]. Written informed consent was obtained from each participant in this study.

#### 4.6.2. Study Design

The study was conducted in a one-sided, blinded, placebo-controlled design with a comparison of untreated skin and skin treated with SDS solution. The measurement of the biophysical parameters of the skin took place in an air-conditioned room (temperature 22–24 °C, relative humidity 45–50%) after twenty minutes of volunteer acclimatization. Both volar sides of the forearm were divided into 10 test sites, each with an area of 8 cm^2^. The first place of the volar side of the forearm remained untreated, which served as the so-called control for visual comparison because of possible skin irritation. The second place also remained without the application of emulsion formulations. These two areas served for the initial measurement (t = 0 h). All other sites were pre-treated with a 0.5 wt% solution of sodium dodecyl sulphate (SDS, Sigma-Aldrich, St. Louis, MO, USA) in saline and left for four hours. After this time, 0.1 mL of each of eight emulsion formulations (B, BS, BG, BL, BM, BMS, BMG, and BML) were applied to one of eight pre-treated sites. The effect of these formulations was monitored after 1, 2, 3, 4, and 24 h.

#### 4.6.3. Non-Invasive Instrumental Bioengineering Methods

The MPA station (Courage & Khazaka Electronic GmbH, Cologne, Germany) was the platform for diagnosing the effect of the prepared emulsion matrices on the skin. To measure the water content in the *stratum corneum*, the Corneometer^®^ CM 825 corneometric probe (Courage & Khazaka Electronic GmbH, Cologne, Germany) was used. It is based on the evaluation of changes in electrical capacitance corresponding to the water content in the *stratum corneum* of the skin. Hydration was measured five times at each marked site of the volar side of the forearm, and the mean value with standard deviation was expressed in corneometric units (c.u.) according to the scale. A scale of < 30 c. u. corresponds to extremely dry skin, values of 30–40 c.u. to dry skin, and normally hydrated skin to values of >40 c.u. [77].

Another tested parameter was TEWL, which was monitored by the Tewameter^®^ TM 300 probe (Courage & Khazaka Electronic GmbH, Cologne, Germany) at each test site 15 times; the first 5 values were neglected due to the equalization of temperature and humidity in the probe chamber and the skin surface of the volunteers’ volar forearm. From the last 10 values, the mean and standard deviation were calculated. In principle, the method determines the flow of water vapor above the s*tratum corneum* into the space of a cylindrical chamber with two pairs of sensors for temperature and relative humidity. TEWL is calculated from the difference between the two measurement points using Fick ’s law of diffusion and displayed in grams per hour per square meter. The interpretation of the results was based on a scale that characterizes the condition of the skin in the range of 0–10 g·m^−2^·h^−1^ for very healthy condition, 10–15 g·m^−2^·h^−1^ for healthy condition, 15–25 g·m^−2^·h^−1^ for normal condition, 25–30 g·m^−2^·h^−1^ for strained skin and above 30 g·m^−2^·h^−1^ for skin in critical condition [78].

To determine the acidity of the skin surface, the skin-pH-meter^®^ PH 905 was used (Courage & Khazaka Electronic GmbH, Cologne, Germany). The specially designed probe consists of a flat-topped glass electrode for full skin contact, connected to a voltmeter. The system measures potential changes due to the activity of hydrogen cations surrounding the very thin layer of semisolid forms at the top of the probe. The changes in voltage are displayed as pH, which has been interpreted as acidic for the range of 3.5–4.3; normal for the range of 4.5–5.5; and high >5.7 [79].

### 4.7. Statistical Analysis and Data Processing

Microbiological results and diagnostically gained biophysical characteristics were statistically analyzed using Microsoft Office Excel (version 10, Microsoft, Santa Rosa, CA, USA) and presented as an arithmetic mean and standard deviation. Subsequently, the values were tested by a paired *t*-test for statistical significance (*p* < 0.05) compared to pre-treatment values in individual time intervals.

The ranking tests were evaluated by the Friedman test, and the paired comparison test was assessed by the Fisher test criterion. The results of the sensory analysis were processed at a 5% significance threshold (*p* < 0.05) by Unistat 5.5 software (Unistat Ltd., London, UK).

## 5. Conclusions

The concept of sustainable natural cosmetics extends beyond the mere formulation of products with natural ingredients. It encompasses a comprehensive strategy to uncover and harness other potential effects of these ingredients. Developing topical products involves not only considering the active ingredients but also ensuring the formulation of a stable and safe product, which poses a significant challenge. In the context of the COVID-19 pandemic, personal care and cosmetic products with antimicrobial and antiviral properties have become indispensable. The emulsion prepared from natural ingredients, fortified with preservatives such as *Litsea Cubeba Oil* and *Mandelic Acid*, demonstrated satisfactory antimicrobial, biophysical, and sensory properties. Incorporating these components into the matrix resulted in reduced viscosity and pH values. The inherent self-preservation capabilities of this system suggest sustainable potential for minimizing or even eliminating the need for traditional preservatives. By adopting such systems, cosmetic products can be labelled as ’preservative-free’, providing consumers with safety assurance.

## Figures and Tables

**Table 1:** Antimicrobial activity of essential oils using the disk diffusion method (diameters of inhibition zones are in mm, including the 6 mm paper disk).

	Gram-Positive Bacteria		Gram-Negative Bacteria				Yeasts
	SA	CuA	EC	PA	KO	KA	CA
Control (water)	6.0 ± 0.1 ^a^	6.0 ± 0.1 ^a^	6.0 ± 0.1 ^a^	6.0 ± 0.1 ^a^	6.0 ± 0.1 ^a^	6.0 ± 0.1 ^a^	6.0 ± 0.1 ^a^
*Satureja Montana* *Oil*	27.7 ± 4.0 ^b^	30.3 ± 4.7 ^b^	25.3 ± 4.2 ^b^	10.0 ± 6.9 ^a^	24.7 ± 2.5 ^b^	25.0 ± 6.1 ^b^	20.3 ± 3.7 ^b^
*Lemongrass* *Oil*	45.3 ± 5.9 ^c^	28.7 ± 5.1 ^b^	17.0 ± 2.6 ^c^	6.0 ± 0.1 ^a^	29.0 ± 8.5 ^b,c^	16.7 ± 3.1 ^b^	22.3 ± 4.1 ^b^
*Litsea Cubeba* *Oil*	33.8 ± 7.6 ^b^	17.8 ± 8.4 ^b^	18.0 ± 0.1 ^c^	6.0 ± 0.1 ^a^	34.7 ± 4.2 ^c^	18.0 ± 3.5 ^b^	6.0 ± 0.1 ^a^

SA—*S. aureus*; CuA—*C. acnes*; EC—*Escherichia coli*; PA—*Pseudomonas aeruginosa*; KO—*Klebsiella oxytoca*; KA—*Klebsiella aerogenes*; CA—*Candida albicans*; control—water. Different letters in the column indicate significant differences among the samples, including the control, against each microorganism (*p* < 0.05).

**Table 2:** Antimicrobial effectiveness test: viable counts (Log CFU/g) of *S. aureus* and *P. aeruginosa* in B/M/EOs samples after 7, 14, and 28 days of storage at room temperature.

Days	7	14	28	7	14	28
	*Staphylococcus aureus*			*Pseudomonas aeruginosa*		
B	3.82	<2.30	<2.30	7.00	7.40	6.73
BS	<2.30	<2.30	<2.30	<2.30	<2.30	<2.30
BG	2.35	<2.30	<2.30	<2.30	<2.30	<2.30
BL	<2.30	<2.30	<2.30	3.99	6.00	5.49
BM	<2.30	<2.30	<2.30	<2.30	<2.30	<2.30
BMS	<2.30	<2.30	<2.30	6.00	6.03	5.68
BMG	<2.30	<2.30	<2.30	3.33	<2.30	<2.30
BML	<2.30	<2.30	<2.30	<2.30	<2.30	<2.30

B—base emulsion; BS—base emulsion with *Satureja Montana Oil*; BG—base emulsion with *Lemongrass Oil*; BL—base emulsion with *Litsea Cubeba Oil*; BM—base emulsion with *Mandelic Acid*; BMS—base emulsion with *Mandelic Acid* and *Satureja Montana Oil*; BMG—base emulsion with *Mandelic Acid* and *Lemongrass Oil*; BML—base emulsion with *Mandelic Acid* and *Litsea Cubeba Oil*.

**Table 3:** Rank sums for the sensory properties of the ranking and paired comparison test of emulsion samples.

Ranking Test	B	BS	BG	BL	BM	BMS	BMG	BML
Spreadability	86	75	62	58	46	47	48	46
Preference	66	63	48	39	70	74	56	52
Paired comparison test	B–BM		BS–BMS		BG–BMG		BL–BML	
Absorbency	6–7		9–4		9–4		6–7	

B—base emulsion; BS—base emulsion with *Satureja Montana Oil*; BG—base emulsion with *Lemongrass Oil*; BL—base emulsion with *Litsea Cubeba Oil*; BM—base emulsion with *Mandelic Acid*; BMS—base emulsion with *Mandelic Acid* and *Satureja Montana Oil*; BMG—base emulsion with *Mandelic Acid* and *Lemongrass Oil*; BML—base emulsion with *Mandelic Acid* and *Litsea Cubeba Oil*.

**Table 4:** Characteristics of model emulsion formulations (B, M, and EOs).

	Base Emulsion Matrix (B)			
**Aqueous phase**	**Ingredients (INCI ^a^)**	**[wt%]**	**Function**	**Supplier**
	*Aqua*	60	Solvent	Tomas Bata University in Zlín (Zlín, Czech Republic)
	*Aloe Barbadensis Extract*	2	Regenerating/Revitalizing/Moisturizing	Kosmetické suroviny Ltd. (Praha, Czech Republic)
	*Glycerin*	4	Humectant	Kosmetické suroviny Ltd. (Praha, Czech Republic)
**Oil phase**	*Helianthus Annuus (Sunflower) Seed Oil*	10	Emollient/Moisturizing/Skin conditioning	Libor Baránek (Uherské Hradiště, Czech Republic)
	*Butyrospermum Parkii*	4	Skin conditioning	Kosmetické suroviny Ltd. (Praha, Czech Republic)
	*Cera Alba*	4	Emollient/Emulsifier	Kosmetické suroviny Ltd. (Praha, Czech Republic)
	*Theobroma Cacao (Cocoa) Seed Butter*	2	Emollient/Protective	Kerfoot Group (Northallerton, United Kingdom)
	*Ricinus Communis Seed Oil*	2	Solvent/Emollient	Míča a Harašta (Praha, Czech Republic)
	Olivoil Avenate Emulsifier^®^ (*Aqua, Glyceryl Oleate, Cetearyl Alcohol, Glyceryl Stearate, Potassium Olivoyl Hydrolyzed Oat Protein*)	12	Emulsifier/Emollient	Kosmetické suroviny Ltd. (Praha. Czech Republic)
	**99.8 wt% B + 0.2 wt% essential oil (EO)**			
	*Satureja Montana Oil*	0.2	Antimicrobial agent	Nobilis Tilia (Krásná Lípa, Czech Republic)
	*Cymbopogon Schoenanthus (Lemongrass) Oil*	0.2	Antimicrobial agent	Nobilis Tilia (Krásná Lípa, Czech Republic)
	*Litsea Cubeba Oil*	0.2	Antimicrobial agent	Saloos (Blansko, Czech Republic)
	**95.0 wt% B + 5 wt% *Mandelic Acid* (M) solution**			
	*Mandelic Acid*	5	Antimicrobial agent	Sigma-Aldrich (St. Louis, MO, USA)
	**94.8 wt% B + 5 wt% *Mandelic Acid* (M) + 0.2 wt% essential oil (EO)**			
	*Satureja Montana Oil*	0.2	Antimicrobial agent	Nobilis Tilia (Krásná Lípa, Czech Republic)
	*Cymbopogon Schoenanthus (Lemongrass) Oil*	0.2	Antimicrobial agent	Nobilis Tilia (Krásná Lípa, Czech Republic)
	*Litsea Cubeba Oil*	0.2	Antimicrobial agent	Saloos (Blansko, Czech Republic)

^a^ INCI (International Nomenclature of Cosmetic Ingredients).

## Data Availability

Data are contained within the article and Supplementary Materials.

## Supplementary Materials

The following supporting information can be downloaded at: https://www.mdpi.com/article/10.3390/molecules29184510/s1, Table S1: Chemical composition of the EOs used (Nobilis Tilia, Czech Republic); Table S2: List of allergens in the EOs used (Nobilis Tilia and Saloos, Czech Republic).
