# Supplementary material for: Formulating Sustainable Emulsions: Mandelic Acid and Essential Oils as Natural Preservatives
Source: Molecules. 2024 Sep 23;29(18):4510. doi: 10.3390/molecules29184510 (PMC11435087; doi:10.3390/molecules29184510)
Supplement: Supplementary file 1 [file molecules-29-04510-s001.zip › molecules-3197160-supplementary.pdf]

Supplementary material to

Formulating Sustainable Emulsions: Mandelic Acid and Essential Oils as Natural Preservatives

Jana Pavlačková [1], Pavlína Egner [1], Pavel Mokrejš [2] and Magda Janalíková [3]

[1] Department of Fat, Surfactant and Cosmetics Technology, Faculty of Technology, Tomas Bata University in Zlín, Vavrečkova 5669, 760 01 Zlín, Czech Republic; pavlackova@utb.cz, egner@utb.cz
[2] Department of Polymer Engineering, Faculty of Technology, Tomas Bata University in Zlín, Vavrečkova 275, 760 01 Zlín, Czech Republic; mokrejs@utb.cz
[3] Department of Environmental Protection Engineering, Faculty of Technology, Tomas Bata University in Zlín, Vavrečkova 275, 760 01 Zlín, Czech Republic; mjanalikova@utb.cz

In this supplementary material, the following tables are presented:

Table S1. Chemical composition of used EOs (Nobilis Tilia, Krásná Lípa, Czech Republic and Saloos, Blansko, Czech Republic).

Table S2. List of allergens in used EOs (Nobilis Tilia, Krásná Lípa, Czech Republic and Saloos, Blansko, Czech Republic).

Table S1. Chemical composition of used EOs (Nobilis Tilia and Saloos, Czech Republic).

Main Components in EO*	Content in EO (%)		
	Satureja Montana	*Lemongrass*	*Litsea Cubeba*
Limonene	-	-	1.0–10.0
α-Pinene	-	-	3.0–9.0
γ-Terpinene	19.5–42.8	-	-
p-Cymene	2.5–0.9	-	-
Carvacrol	15.0–70.7	-	-
Geranial	-	< 42.0	45.0–50.0
Geraniol	-	< 1.5	-
Neral	-	< 40.0	5.0–65.0

*Total allergens up to 0.001%

Table S2. List of allergens in used EOs (Nobilis Tilia and Saloos, Czech Republic).

**Allergen List in EO**	**CAS Nr.**	**Content in EO (%)**		
		Satureja Montana	***Lemongrass***	***Litsea Cubeba***
Citral	5392-40-5	-	60.0–82.0	50.0–80.0
Citronellol	106-22-9	-	-	< 1.0
Geraniol	106-24-1	-	< 1.5	< 3.0
Limonene	5989-27-5	< 1.0	< 3.0	1.0–10.0
Linalool	78-70-6	-	< 2.0	< 5.0
